# Elevated preoperative peripheral blood monocyte count predicts poor prognosis for hepatocellular carcinoma after curative resection

**DOI:** 10.1186/1471-2407-14-744

**Published:** 2014-10-03

**Authors:** Shun-Li Shen, Shun-Jun Fu, Xiong-Qing Huang, Bin Chen, Ming Kuang, Shao-Qiang Li, Yun-Peng Hua, Li-Jian Liang, Bao-Gang Peng

**Affiliations:** Department of Hepatobiliary Surgery, The First Affiliated Hospital, Sun Yat-sen University, Guangzhou, 510080 China; Department of Hepatopancreatobiliary Surgery, The Scecond Affiliated Hospital of Guangzhou University of Chinese Medicine, Guangzhou, 510120 China; Department of Anesthesiology, The First Affiliated Hospital, Sun Yat-sen University, Guangzhou, 510080 China

**Keywords:** Hepatocellular carcinoma, Monocyte, Hepatic resection, Prognosis

## Abstract

**Background:**

Peripheral blood monocyte count is an easily assessable parameter of systemic inflammatory response. The aim of this study was to determine whether monocyte count was prognostic in hepatocellular carcinoma (HCC) following hepatic resection.

**Methods:**

We retrospectively reviewed 351 patients with HCC treated with hepatic resection from 2006 to 2009. Preoperative absolute peripheral monocyte count, demographics, and clinical and pathological data were analyzed.

**Results:**

On univariate and multivariate analysis, elevated monocyte counts (≥545/mm^3^), tumor size ≥5 cm, non-capsulation, and multiple tumors were associated with poor disease-free survival (DFS) and overall survival (OS). The 1-, 3- and 5-year DFS rates were 58%, 41% and 35%, respectively, for patients with monocyte counts <545/mm^3^, and 36%, 23% and 21% for patients with monocyte counts ≥545/mm^3^. Correspondingly, the 1-, 3- and 5-year OS rates were 79%, 53% and 46% for monocyte counts <545/mm^3^, and 64%, 36% and 29% for monocyte counts ≥545/mm^3^. Subgroup analysis indicated that DFS after hepatic resection in hepatitis B virus (HBV)-infected patients was significantly better in those with a peripheral blood monocyte counts <545/mm^3^, but it did not differ between patients without HBV infection. In addition, DFS was significantly better for patients with a peripheral blood monocyte count <545/mm^3^, whether or not cirrhosis was present. Patients with elevated monocyte counts tended to have larger tumors.

**Conclusions:**

Elevated preoperative monocyte count is an independent predictor of worse prognosis for patients with HCC after hepatic resection, especially for those with HBV infection. Postoperative adjuvant treatment might be considered for patients with elevated preoperative monocyte counts.

## Background

Hepatocellular carcinoma (HCC) is the fifth most common cancer and the third most frequent cause of cancer-related death [1]. Hepatic resection is one of the most effective treatments for HCC. However, even after hepatic resection, the long-term prognosis has remained far from satisfactory because of the high rate of recurrence.

Therapies such as re-operation, percutaneous ablation, and transcatheter arterial chemoembolization (TACE) might be considered for recurrent HCC [2–4]. Unfortunately, however, there are limited or no therapeutic options for a large number of patients when recurrence is found. It is therefore critical to define reliable prognostic factors that may help identify patients at high risk of recurrence. In addition, this group of patients might benefit from postoperative adjuvant therapy against recurrence [3, 4].

Prognostic factors identified in previous studies include tumor stage, serum α-fetoprotein (AFP), vascular invasion, tumor size, and poor tumor differentiation [2]. Novel immunological and histological biomarkers have also been identified, but they tend to be time consuming to measure and do not constitute standard practice. Emerging evidence indicates that peripheral blood cells reflect the inflammatory status of patients, and they are predictors of prognosis in cancer patients. The association between pretreatment peripheral leukocytes (including neutrophils, lymphocytes and platelets) and prognosis has been observed in various cancers, including colon cancer, melanoma and pancreatic carcinoma. In addition, systemic-inflammation-based scores such as Glasgow prognostic score (GPS), neutrophil lymphocyte ratio (NLR), and platelet lymphocyte ratio (PLR) have prognostic value in cancer patients [5].

Regarding HCC, preoperative elevated NLR is associated with short disease-free survival (DFS) and overall survival (OS) after hepatectomy or liver transplantation [6, 7]. Likewise, increased C-reactive protein or monocyte count has been linked with poor prognosis [8, 9]. Monocytes play an important role in innate immunity and exhibit prognostic value in cancers. High pretreatment monocyte count is an independent factor of poor prognosis for patients with colorectal liver metastasis, cervical cancer, melanoma, and HCC [9–12]. In fact, circulating monocytes predict incident cancer and mortality even in healthy individuals [13].

The aim of the present study was to evaluate whether elevated preoperative peripheral monocyte count predicts poor prognosis in HCC patients after hepatic resection, especially in hepatitis B virus (HBV)-associated HCC.

## Methods

### Study population

We enrolled 351 patients with newly diagnosed HCC treated with hepatectomy at The First Affiliated Hospital, Sun Yat-Sen University, Guangdong, China between 2006 and 2009. All specimens were histologically proven to be HCC after surgery. The routine workup was done within 7 days before surgery, which included a complete physical examination, hematological and biochemistry profiles, abdominal ultrasound and computed tomography (CT) or magnetic resonance imaging (MRI), chest X-ray or CT scan. Final diagnosis of HCC was made by pathological examination of biopsy specimens. All patients were >18 years of age, with complete clinical and laboratory data. No patients had any coexistent hematological disorders or known active infection before treatment, ensuring that the monocyte count was representative of the normal baseline value. In addition, patients with mixed HCC and cholangiocarcinoma and patients with no follow-up data were excluded. Informed consent was obtained, and procedures were carried out with prior approval of the Ethics Committee of the First Affiliated Hospital of Sun Yat-sen University (Guangzhou, China).

### Treatment and follow-up

Hepatectomy was performed on all patients with intent to cure, and tumor thrombectomy or combined diaphragmatic resection was carried out when necessary. Surgical resection was defined as radical when there was no evidence of distant metastases and tumor clearance was both macroscopically and histologically complete. Patients were regularly followed up at outpatient clinics every 3 months for the first 2 years, every 6 months for the next 3 years, and once annually thereafter. Patients received a physical examination, liver ultrasound, chest X-ray and AFP test at each follow-up. Abdominal CT scan was performed every 6–12 months or when recurrence was suspected. Recurrence was defined as emergence of clinical, radiological, and/or pathological diagnosis (tissues obtained by ultrasound-guided fine-needle aspiration) of tumor. Once recurrence was confirmed, salvage treatments including re-operation, percutaneous ablation (ethanol injection, radiofrequency ablation, or microwave ablation) or TACE were selected as needed.

### Statistical analysis

Statistical analysis was performed using SPSS for Windows version 16.0 (SPSS, Chicago, IL, USA). Receiver operating characteristic (ROC) curve analysis was performed to select the most appropriate cut-off value of monocyte count to stratify patients at a high risk of tumor recurrence. At each value, sensitivity and specificity were plotted, thus generating an ROC curve. The score closest to the point with both maximum sensitivity and specificity was selected as the cut-off value. The χ^2^ test was used to compare categorical variables. DFS and OS were calculated from the date of surgery to the date of recurrence, or HCC-associated death, respectively. Survival curves were plotted using the Kaplan–Meier method and compared using the log-rank test. The Cox proportional hazards model was used to determine independent prognostic factors on the basis of variables selected on univariate analysis. *P* < 0.05 was considered significant.

## Results

### Patient and tumor characteristics

There were 310 (88.3%) male and 41 (11.7%) female patients. The mean age of patients was 50.1 years (range: 21–79 years). Two hundred and fifty-six patients (72.9%) developed recurrence while 222 (63.2%) died during follow-up. Hepatitis B surface antigen (HBsAg) was positive in 302 patients (86.0%) and 269 (76.6%) patients had underlying cirrhosis. Increased AFP (>200 ng/ml) was found in 190 cases (54.1%) and 75 (21.4%) patients had ≥2 tumors in the liver. Mean tumor size (greatest dimension) was 89.0 mm (range: 10–300 mm), with 281 (80.1%) patients having tumors >50 mm. With regard to tumor differentiation according to Edmonson stage, there were 271 (77.2%) I/II stage and 80 (22.8%) III/IV stage tumors. Macroscopic vessel invasion into the portal vein, hepatic vein or inferior vena cava was found in 90 patients (25.6%). Details of features are shown in Table 1.

**Table 1:** **Comparison of clinicopathological features of patients with different monocyte counts**

Category	Subcategory	Cases	Monocytes(/mm ^3^)		P value
			<545	≥545	
**Gender**	Male	310	115	195	0.102
	Female	41	20	21	
**Age**	<50 years	268	102	166	0.418
	≥50 years	83	33	50	
**HBsAg**	Negative	49	14	35	0.083
	Positive	302	121	181	
**AFP** **(ng/** **ml)**	<200	161	61	100	0.463
	≥200	190	74	116	
**Edmonson grading**	I-II	271	107	164	0.278
	III-IV	80	28	52	
**Tumor size**	<5 cm	70	35	35	0.019
	≥5 cm	281	100	181	
**Surgical margin**	<1 cm	122	44	78	0.289
	≥1 cm	229	91	138	
**Liver cirrhosis**	Absent	82	29	53	0.3
	Present	269	106	163	
**PVTT**	Absent	278	108	170	0.44
	Present	73	27	46	
**Capsulation**	Capsulated	224	92	132	0.111
	Non-caspulated	127	43	84	
**Tumor number**	Single	276	107	169	0.465
	Multiple	75	28	47	
**Macrovascular invasion**	Absent	261	102	159	0.391
	Present	90	33	57	

### Relationship between clinicopathological features and monocyte status

**Figure 1:**
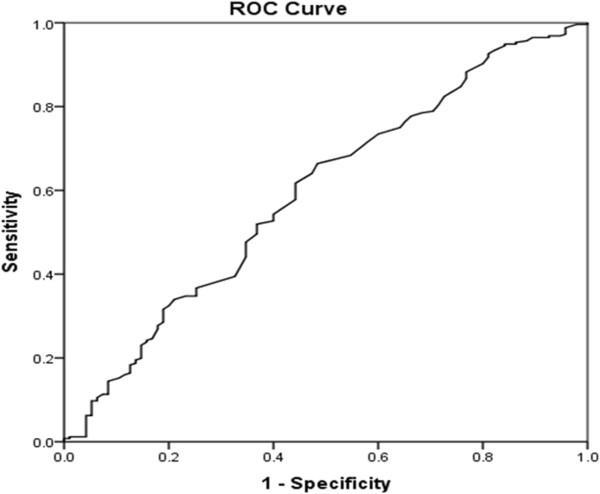
**Receiver operating characteristic curve for determination of the cut-off value for monocyte in patients with hepatocellular carcinoma (HCC) after hepatic resection.**

The median monocyte count was 600/mm^3^, which was almost twice as high as the standard used by Sasaki et al. [9]. To exclude empirical bias, we used ROC curve analysis to determine the optimal cut-off value for elevated monocyte count. A cut-off value of 545/mm^3^ corresponded to the maximum joint sensitivity and specificity on the ROC plot (Figure 1). Clinicopathological features of patients with different monocyte status are summarized in Table 1. None of the commonly used clinicopathological features (age, gender, Edmonson grade, HBsAg status, surgical margin, capsulation, tumor number and cirrhosis) were significantly different between the two groups. However, patients with elevated monocyte counts tended to have larger tumors (*P* = 0.019).

### Risk factors for prognosis of HCC after hepatectomy

For all patients, the 1-, 3- and 5-year DFS was 44%, 30% and 27%, respectively, and the 1-, 3- and 5-year OS was 70%, 43%, 36%, respectively. Univariate analysis (Table 2) revealed that macrovascular infiltration, monocyte counts ≥545/mm^3^, surgical margin <1 cm, AFP ≥200 ng/ml, tumor size ≥5 cm, non-capsulation (tumor had no capsulation or ruptured during surgery), multiple tumors, and macrovascular invasion were associated with significantly poorer DFS. Similarly, monocyte counts ≥545/mm^3^, surgical margin <1 cm, tumor size ≥5 cm, non-capsulation, and multiple tumors predicted poor OS.

**Table 2:** **Univariate and multivariate analysis of clinicopathological parameters influencing prognosis**

Category	Subcategory	Disease-free survival			Overall survival		
		Univariate analysis	Multivariate analysis	HR(95% CI)	Univariate analysis	Multivariate analysis	HR(95% CI)
**Gender**	Male	0.25			0.064		
	Female						
**Age**	<50 years	0.079			0.078		
	≥50 years						
**HBs**-**Ag**	Negative	0.412			0.978		
	Positive						
**AFP** **(ng/** **ml)**	<200	0.021			0.209		
	≥200						
**Edmonson grading**	I-II	0.277			0.788		
	III-IV						
**Tumor size**	<5 cm	< 0.001	< 0.001	1.991 (1.383-2.867)	0.001	< 0.001	1.919 (1.291-2.582)
	≥5 cm						
**Surgical margin**	<1 cm	0.001			0.002		
	≥1 cm						
**Liver cirrhosis**	Absent	0.505			0.91		
	Present						
**Monocyte count** **(/mm** ^**3**^ **)**	<545	< 0.001	0.01	1.393 (1.083-1.793)	< 0.001	0.002	1.578 (1.187-2.097)
	≥545						
**PVTT**	Absent	0.545			0.911		
	Present						
**Capsulation**	Capsulated	< 0.001	< 0.001	1.597 (1.231-2.071)	0.002	< 0.001	1.793 (1.371-2.345)
	Non-caspulated						
**Tumor number**	Single	< 0.001	0.005	1.513 (1.134-2.019)	< 0.001	< 0.001	1.771 (1.312-2.392)
	Multiple						
**Macrovascular invasion**	Absent	0.012			0.086		
	Present						

*CI*, confidence interval; *HR*, hazard ratio.

On multivariate analysis, four parameters including monocyte counts ≥545/mm^3^, tumor size ≥5 cm, non-capsulation, and multiple tumors were independent prognostic factors of poor DFS and OS.

### DFS and OS according to monocyte status

**Figure 2:**
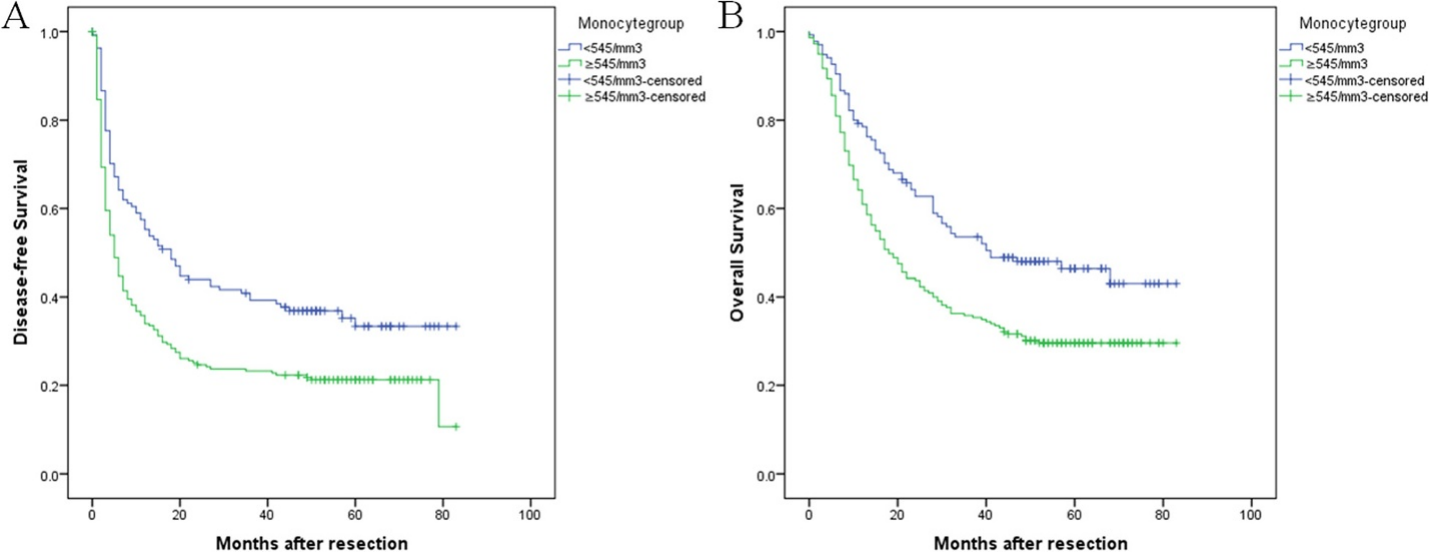
**Disease-free survival and overall survival of 351 HCC patients after hepatectomy with different monocyte.** The patients were divided into monocyte ≥ 545/mm^3^ and monocyte < 545/mm^3^ group by the optimal cut-off value of monocyte. **(A)** Disease-free survival of patients with monocyte ≥ 545/mm^3^ was shorter than those with monocyte < 545/mm^3^ (*P* < 0.001, log-rank). **(B)** Overall survival of patients with monocyte ≥ 545/mm^3^ was also shorter than those with monocyte < 545/mm^3^ (*P <* 0.001, log-rank).

The 1-, 3- and 5-year DFS rate was 58%, 41% and 35% for patients with monocyte counts <545/mm^3^, and 36%, 23% and 21% for patients with monocyte counts ≥545/mm^3^, respectively (*P* < 0.001). Correspondingly, the 1-, 3- and 5-year OS rate was 79%, 53% and 46% for patients with monocyte counts <545/mm^3^, and 64%, 36%, 29% patients with monocyte counts ≥545/mm^3^. Both DFS and OS of patients with monocyte counts <545/mm^3^ were significantly better than for patients with monocyte counts ≥545/mm^3^ (*P* < 0.001) (Figure 2).

### Subgroup analysis according to the HBV infection and cirrhosis status

**Figure 3:**
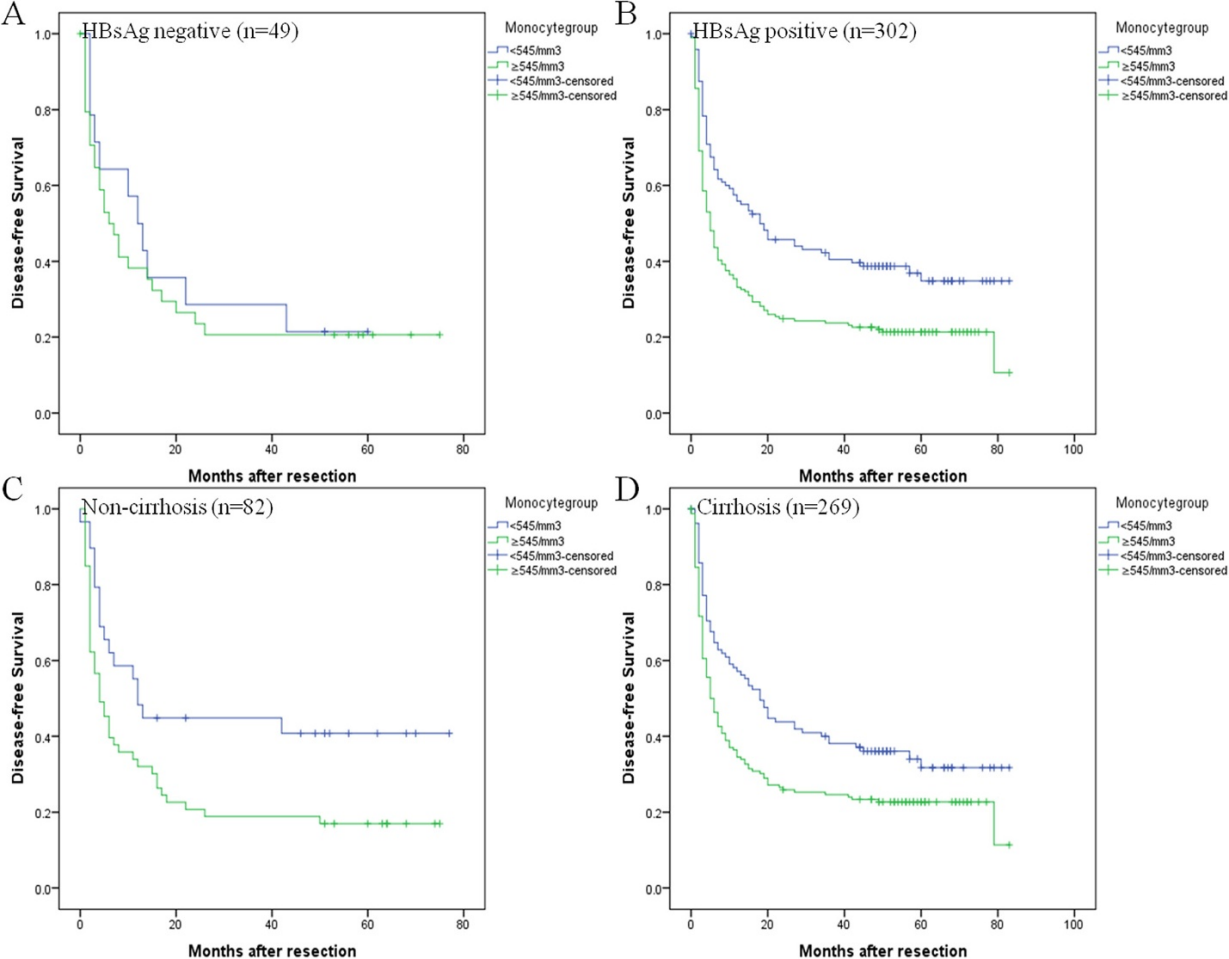
**Subgroup analysis of disease-free survival of 351 HCC patients after hepatectomy with different monocyte based on HBV or cirrhosis status. (A)** monocyte count could not separate patients with different DFS rates in patients with HBV negative group (*P* = 0.858). **(B)** By contrast, monocyte count predicted different DFS rates in patients with HBV positive group (*P* < 0.001). **(C)** In addition, DFS was significantly better for the patients with a peripheral blood monocyte count < 545/mm^3^ than those with monocyte ≥ 545/mm^3^ in non-cirrhosis group (*P* = 0.018). **(D)** DFS was also significantly better for the patients with a peripheral blood monocyte count < 545/mm^3^ than those with monocyte ≥ 545/mm^3^ in cirrhosis group (*P* = 0.002).

To clarify the subgroups of patients negatively influenced by preoperative peripheral blood monocyte counts, we classified patients according to accompanying liver disease (HBsAg positive, *n* = 302; HBsAg negative, *n* = 49) and underlying cirrhosis (cirrhosis, *n* = 269; non-cirrhosis, *n* = 82). DFS after hepatic resection of HBV-infected patients was significantly better for those with a peripheral blood monocyte count <545/mm^3^ (*P* < 0.001), but DFS did not differ between patients without HBsAg infection within groups (*P* = 0.607). In contrast to the results of Sasaki et al., we found that DFS was significantly improved for the patients with a peripheral blood monocyte count <545/mm^3^, whether or not cirrhosis was present (*P* = 0.002 vs *P* = 0.018) (Figure 3).

## Discussion

We demonstrated that elevated monocyte count independently predicted worse survival in HCC patients treated with hepatic resection, which concurs with a previous study by Sasaki et al. [9]. In addition, tumor size ≥5 cm, non-capsulation, and multiple tumors were also independent prognostic factors of poor DFS and OS.

Numerous clinical and experimental studies have indicated that inflammation is a critical component of tumor progression. Inflammatory markers such as C-reactive protein have been suggested as surrogate markers for HCC [8]. Likewise, subsets of peripheral blood cells have been found to be predictors of prognosis. Elevated NLR was shown to be an indicator of poor outcome in patients undergoing hepatic resection for colorectal liver metastasis and curative resection for HCC [14, 15]. An elevated neutrophil, monocyte or leukocyte count was associated with poor survival in patients with metastatic melanoma [12]. In addition, a higher pretreatment circulating monocyte count was independently associated with poor prognosis in patients with locally advanced cervical squamous cell carcinoma and HCC [9, 10]. However, in all of these studies, the cut-off value for monocyte count was based on a median value of circulating monocyte count [10]. In the present study, the median monocyte count was two times higher than that of Sasaki et al. To exclude empirical bias, we used ROC curve to determine the optimal cut-off value to predict HCC recurrence after hepatectomy.

Monocyte count can be easily measured by routine preoperative blood workup and is a strong prognostic factor for a number of malignancies, such as colorectal cancer with liver metastasis and melanoma. With regard to HCC, Sasaki et al. first reported that monocyte count was a useful prognostic indicator in HCC patients [9]. In that study, a preoperative absolute peripheral blood monocyte count >300/mm^3^ was shown to be an independent prognostic indicator of tumor recurrence, especially in patients with HCC accompanying liver cirrhosis [9]. In their series, serum hepatitis C antibody was positive in 100 (65.4%) of the 153 tested patients and HBV infection was positive in only 23.74% (47/198) patients, which was different from our data (86%, 302/351 patients). From subgroup analysis we found that elevated monocyte predicted early recurrence whether or not cirrhosis was present, which was different from the study of Sasaki et al. Patients in these two studies have a different background of cirrhosis, which might account for the difference. In addition, we found that elevated monocyte count predicted poor prognosis in HBV-positive HCC patients, but not in negative ones. This is a novel finding.

The present study confirms that preoperative monocyte count is an independent prognostic factor for HCC, especially in patients with an HBV background. After surgery, too many factors influence postoperative peripheral blood monocyte count, such as bleeding, shortage or overuse of liquid replacement, and sepsis. Therefore, postoperative peripheral blood inflammatory cells have not been used often to predict prognosis as the preoperative counterparts, although there are a few such reports [16–18]. In fact, in the study of Lee et al., monocyte count significantly increased after surgery, but the authors did not detect significant effects on circulating monocytes and survival [18]. We think it might be unsuitable to use postoperative monocyte count to predict prognosis for too many confounding factors.

The exact pathophysiology for the association between high monocyte counts and poor prognosis is not well understood. There are several possible explanations. First, it has been hypothesized that activation of the innate immune system through mobilization of monocytes to tissue macrophages develops an inflammatory state associated with increased risk of cancer and mortality [19–21]. Tumor-associated macrophages (TAMs), which arise from blood monocytes, appear to play a crucial role in the tumor microenvironment and can educate and control invading leukocytes to promote angiogenesis, viability, motility and invasion [19–21]. Monocytes are actively attracted to the tumor site and differentiate into TAMs as a result of the production of cytokines and chemokines by tumor cells, such as monocyte chemoattractant protein-1 [or chemokine CC ligand (CCL2)], RANTES (or CCL5) and vascular endothelial growth factor. TAMs are not only capable of killing tumor cells and releasing angiostatic compounds, but can exert pro-tumor effects through the secretion of immunosuppressive cytokines and angiogenic factors. Unfortunately, the pro-tumor effects of TAMs often outweigh the tumor-inhibiting effects during tumor development. The number of TAMs has been shown to correlate with poor prognosis [19–22]. Second, elevated CD14^+^CD16^+^ monocytes (a minor blood monocyte subpopulation) correlate with TAM infiltration. These monocytes express higher levels of adhesion molecules and scavenger receptors, which enable them to adhere to endothelial cells, and they also express high levels of growth-factor- and angiogenic-factor-related genes. All these characteristics indicate that CD14^+^CD16^+^ monocytes have protumorigenic features and might be associated with rapid tumor progression and poor patient outcome [23]. Third, a fraction of monocytes/macrophages in peritumoral stroma expresses surface programmed death ligand (PD-L)1 molecules in tumors from patients with HCC. The PD-L1^+^ monocytes effectively suppress tumor-specific T cell immunity and contribute to the growth of human tumors in vivo, which can be reversed by blocking PD-L1 on these monocytes. Moreover, PD-L1 expression on tumor-infiltrating monocytes is increased with disease progression, and the intensity of the protein is associated with high mortality and reduced survival in HCC patients. Thus, expression of PD-L1 on activated monocytes/macrophages may represent a novel mechanism that links the proinflammatory response to immune tolerance in the tumor milieu [24]. In short, monocytes might contribute to the compromised antitumor microenvironment, thus promoting tumor progress.

HCC shows low responsiveness to standard chemotherapeutic agents or radiotherapy, like other tumors (such as osteosarcoma, lung cancer, and breast cancer). Therefore, a high preoperative monocyte count does not necessitate neoadjuvant therapy [25–27]. Instead, postoperative adjuvant therapy might be considered. Nevertheless, for HCC patients after curative resection, there is no consensus on the use of adjuvant therapy outside of clinical trials [3]. However, studies have shown that HCC patients with a high risk of recurrence are likely to benefit from postoperative adjuvant treatment such as chemotherapy, TACE, or antiviral therapy [28, 29]. A study from Xia et al. [30] showed that adjuvant therapy with capecitabine postponed recurrence of HCC after curative resection. In addition, an adjuvant intraportal venous chemotherapy regimen of cisplatin, interferon-α, doxorubicin, and 5-fluorouracil (PIAF) for HCC patients with portal vein tumor thrombus (PVTT) following hepatectomy, plus portal thrombectomy, significantly delayed recurrence and prolonged survival [31]. Risk of HCC recurrence after potentially curative resection was higher in the setting of high viral replication and ongoing inflammatory activity in the liver. In a meta-analysis, antiviral therapy with interferon was found to improve 1-, 2- and 3-year recurrence-free survival by 7.8%, 35.4% and 14.0%, respectively (all *P* < 0.01) [32]. Likewise, oral antiviral drugs (including lamivudine, adefovir and entecavir) showed potential beneficial effects after curative treatment of HBV-related HCC in terms of tumor recurrence, liver-related mortality, and OS [33, 34]. Although it remains unclear whether TACE actually decreases the risk of tumor recurrence, it has been reported that postoperative TACE prevents early recurrence, while antiviral therapy prevents late recurrence of HCC. Combination of antiviral therapy and TACE is suggested for prevention in HCC patients at high risk of recurrence [28, 29, 35]. Multicenter studies evaluating the effects of adjuvant kinase inhibitor treatments with sorafenib after curative resection or tumor ablation are currently underway (STORM study from Bayer) [4]. Until the results of these studies are available, the role of adjuvant or neoadjuvant treatments with kinase inhibitors in the prevention of tumor recurrence in the setting of potentially curative treatments for HCC remains unknown.

There were two limitations to our study. First, we were not able to split our data set into a training data set and a test data set for statistical validation because of the small number of patients, which we hope to validate in future studies, or from other centers. Second, although we found that elevated monocyte count predicted early recurrence, and that these patients might benefit from postoperative adjuvant therapy, we were not able to test this hypothesis, which we hope to prove in future clinical trials.

## Conclusions

Our results show that the absolute number of peripheral blood monocytes may be related to tumor progression and is an independent risk factor for recurrence of HCC after hepatic resection, especially for patients with HBV infection. Future clinical trials to test the efficacy of postoperative adjuvant treatment in HCC patients with an elevated preoperative monocyte count might be considered.

## Abbreviations

AFP: α-fetoprotein
CT: Computed tomography
DFS: Disease-free survival
GPS: Glasgow prognostic score
HBV: Hepatitis B virus
HCC: Hepatocellular carcinoma
MRI: Magnetic resonance imaging
NLR: Neutrophil lymphocyte ratio
OS: Overall survival
PIAF: Cisplatin, interferon-α, doxorubicin, and 5-fluorouracil
PLR: Platelet lymphocyte ratio
PVTT: Portal vein tumor thrombus
ROC: Receiver operating characteristic
TACE: Transcatheter arterial chemoembolization
TAM: Tumor-associated macrophage.
